# Photosystem I Inhibition, Protection and Signalling: Knowns and Unknowns

**DOI:** 10.3389/fpls.2021.791124

**Published:** 2021-12-01

**Authors:** Yugo Lima-Melo, Mehmet Kılıç, Eva-Mari Aro, Peter J. Gollan

**Affiliations:** ^1^Post-graduation Programme in Cellular and Molecular Biology (PPGBCM), Department of Botany, Federal University of Rio Grande do Sul, Porto Alegre, Brazil; ^2^Molecular Plant Biology, Department of Life Technologies, University of Turku, Turku, Finland

**Keywords:** PSI, photoinhibition, P700, electron transport, ROS, metabolism, photoprotection, alternative electron flow

## Abstract

Photosynthesis is the process that harnesses, converts and stores light energy in the form of chemical energy in bonds of organic compounds. Oxygenic photosynthetic organisms (i.e., plants, algae and cyanobacteria) employ an efficient apparatus to split water and transport electrons to high-energy electron acceptors. The photosynthetic system must be finely balanced between energy harvesting and energy utilisation, in order to limit generation of dangerous compounds that can damage the integrity of cells. Insight into how the photosynthetic components are protected, regulated, damaged, and repaired during changing environmental conditions is crucial for improving photosynthetic efficiency in crop species. Photosystem I (PSI) is an integral component of the photosynthetic system located at the juncture between energy-harnessing and energy consumption through metabolism. Although the main site of photoinhibition is the photosystem II (PSII), PSI is also known to be inactivated by photosynthetic energy imbalance, with slower reactivation compared to PSII; however, several outstanding questions remain about the mechanisms of damage and repair, and about the impact of PSI photoinhibition on signalling and metabolism. In this review, we address the knowns and unknowns about PSI activity, inhibition, protection, and repair in plants. We also discuss the role of PSI in retrograde signalling pathways and highlight putative signals triggered by the functional status of the PSI pool.

## Introduction

Photosynthesis, the primary source of oxygen and organic compounds, is vital for life on Earth. Photosynthetic activity in plants is intrinsically associated with productivity and yield (Raines, 2011) through allocation of assimilated carbon and biomass accumulation. Therefore, efficient photosynthesis is essential to the problem of boosting crop growth and productivity that is required to match increasing food and fuel demands by the growing global population (Fischer and Edmeades, 2010; Ray et al., 2012; Long et al., 2015; Simkin et al., 2017). Accumulating evidence supports an increase in photosynthetic capacity as a viable route to increase the yield of crop plants (Long et al., 2015; Kromdijk et al., 2016; von Caemmerer and Furbank, 2016; Simkin et al., 2017; Salesse-Smith et al., 2018).

Although the study of photosynthesis is a pillar of the plant sciences, many questions remain concerning its regulation, and how photosynthetic activity influences other processes within the cell and throughout the organism. Unlike decades of extensive research on the damage and repair of photosystem II (PSII) (e.g., Aro et al., 1993; Zavafer and Mancilla, 2021), outstanding questions relating to damage and repair of PSI, the other light-harnessing reaction centre protein complex of the thylakoid membrane, have been less well-studied. Beside PSI protection and inactivation, the impact of PSI inactivation on chloroplast metabolism and retrograde signalling have remained poorly understood. However, absorbance measurements of P700, the special chlorophylls at the PSI reaction centre, are now commonly used to assess PSI quantum yield and electron transport reactions involving PSI (Klughammer and Schreiber, 2008, 2016; Schreiber and Klughammer, 2016), which has improved the understanding of factors regulating PSI activity and/or inactivation.

Here we review the current knowns and unknowns about PSI activity, inhibition, protection, and repair in plants. We also discuss the role of PSI in retrograde signalling pathways and highlight putative signals triggered by the functional status of the PSI pool. Considering the importance of understanding PSI metabolism and regulation, new directions for PSI research are suggested.

## Photosystem I Electron Transport Activity Powers Carbon Metabolism

**FIGURE 1:**
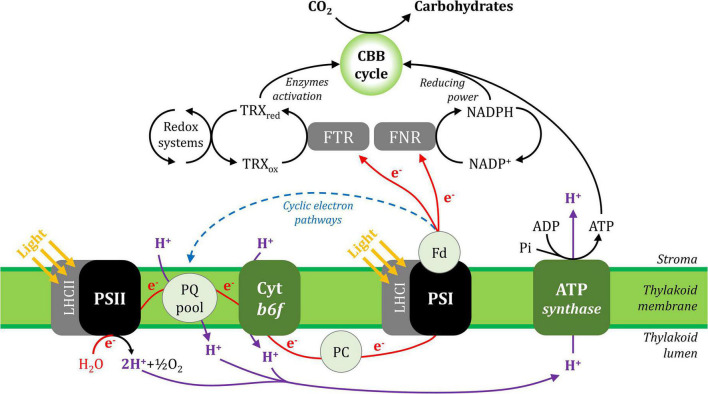
A simplified scheme of the photosynthetic electron transport chain in the thylakoid membrane and its interaction with CO_2_ assimilation in the Calvin-Benson-Bassham cycle. Linear electron (e^–^) transport is shown with red lines and cyclic electron transport is represented with a blue dashed line. The proton (H^+^) fluxes are represented in purple lines. ADP, adenosine diphosphate; ATP, adenosine triphosphate; CBB cycle, Calvin-Benson-Bassham cycle; Cyt *b6f*, cytochrome *b6f*; Fd, ferredoxin; FNR, ferredoxin:NADP^+^ oxidoreductase; FTR, ferredoxin:thioredoxin reductase; LHCI, light-harvesting complex I; LHCII, light-harvesting complex II; NADP^+^, oxidised nicotinamide adenine dinucleotide phosphate; NADPH, reduced nicotinamide adenine dinucleotide phosphate; PC, plastocyanin; Pi, inorganic phosphate; PQ, plastoquinone; PSI, photosystem I; PSII, photosystem II; TRX_ox_, oxidised thioredoxin; TRX_red_, reduced thioredoxin.

In general, photosynthesis converts light energy into chemical energy, which is stored as carbohydrate molecules synthesised from carbon dioxide (CO_2_) and water. In plants, photosynthesis is often separated into two distinct processes; photochemistry and CO_2_ assimilation/fixation, although these steps are inter-related. During photochemistry, chlorophyll and other photosynthetic pigments absorb light energy that is used to extract electrons from water in the lumen and transport them through the thylakoid membrane to reduce the oxidised form of nicotinamide adenine dinucleotide phosphate (NAD^+^), producing its reduced form (NADPH) in the stroma. This process also generates a proton gradient across the thylakoid membrane that produces the energy carrier molecule adenosine triphosphate (ATP). During CO_2_ assimilation, ATP and NADPH generated from the photochemical phase are used to reduce CO_2_ molecules to produce carbohydrates and their derivative products. These processes are shown in Figure 1 and its animated version in the Supplementary Material (Supplementary Video 1).

**FIGURE 2:**
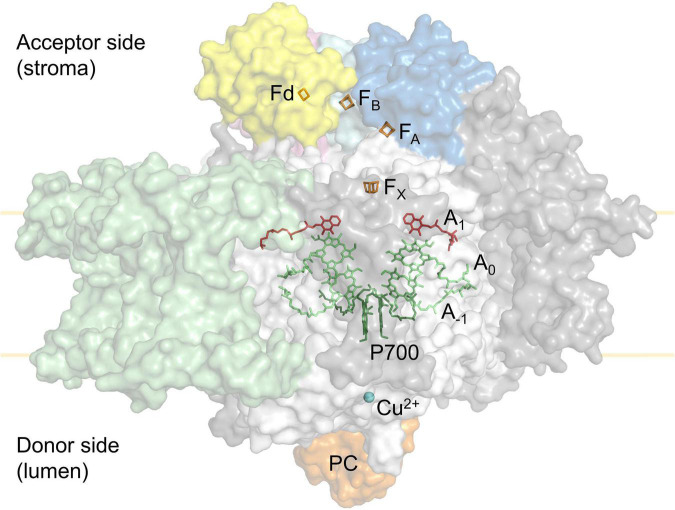
Simplified tertiary structure of the PSI:plastocyanin:ferredoxin complex (PDB accession 6YEZ; Caspy et al., 2020), showing protein subunits PsaA and PsaB (white), PsaC (cyan), PsaD (blue), PsaE (pink) and LHCI subunits (green). Other PSI subunits are coloured grey. Also shown are plastocyanin (orange) at the PSI donor side and ferredoxin (yellow) at the PSI acceptor side. Cofactors involved in electron transport are shown; Cu^2+^ (blue), P700 (dark green), A_–1_ and A_0_ chlorophylls (lime), A_1_ phylloquinone (red) in PSI, as well as the PSI 4Fe4S clusters F_X_, F_A,_ F_B_ (orange) and the ferredoxin (Fd) 2Fe2S cluster (orange).

Linear electron flux begins with water-splitting at PSII and proceeds through sequential reduction and oxidation of cofactors within the thylakoid membrane (plastoquinone; PQ), the cytochrome *b6f* complex (cyt *b6f*), and the thylakoid lumen (plastocyanin; PC), before arriving at the donor side of PSI. Electron transport upstream of PSI will not be detailed here, but has been described in excellent reviews (Freeman and Guss, 2011; Borisova-Mubarakshina et al., 2019; Havaux, 2020; Malone et al., 2021; Sarewicz et al., 2021; Shevela et al., 2021). In the light, the PSI reaction centre receives excitation from both light-harvesting complex I (LHCI), which serves only PSI, and light-harvesting complex II (LHCII), which serves both PSI and PSII (Grieco et al., 2012, 2015; Wientjes et al., 2013; Rantala and Tikkanen, 2018). Excitation promotes PSI charge separation, whereby an electron is ejected from P700 via the monomeric form of chlorophyll *a* named A_0_ and phylloquinone A_1_ to the first iron-sulphur (FeS) cluster F_X_. Cofactors P700, A_0_, A_1_, and F_X_ are bound to the PSI protein subunits PsaA and/or PsaB, which form the central protein heterodimer of PSI and bind the majority of the other subunits of the complex (Figure 2; Golbeck, 1992; Ben-Shem et al., 2003; Amunts et al., 2007; Amunts and Nelson, 2009; Qin et al., 2015; Kozuleva and Ivanov, 2016; Mazor et al., 2017). The electron hole formed by charge separation at P700 is filled from the PSI donor side by oxidation of reduced PC (detailed in Caspy et al., 2021). Electron flux through PSI terminates at the F_A_ and F_B_ clusters housed by the stromal PSI subcomplex PsaC, PsaD, and PsaE at the PSI acceptor side, where there also resides a docking site for oxidised ferredoxin (Fd). PsaC establishes close contact required for fast electron transfer between the respective FeS clusters of PSI and Fd, while PsaD and PsaE are responsible for guidance of Fd into the PSI binding pocket (Busch and Hippler, 2011; Marco et al., 2018; Caspy et al., 2020). Fd reduced by PSI primarily carries electrons to the Fd-NADP^+^-oxidoreductase (FNR) enzyme, which is responsible for producing reduced NADPH that powers the electron-consuming reactions of the chloroplast (reviewed in Hanke and Mulo, 2013). Fd also delivers electrons to the thioredoxin network of the chloroplast, which regulates the redox-dependent activity of CO_2_ assimilation enzymes of the Calvin-Benson-Bassham (CBB) cycle (Buchanan, 2016; Nikkanen et al., 2016). Under specific conditions, reduced Fd also injects electrons back into the PQ pool via cyclic electron transport (reviewed in Peltier et al., 2016).

ATP and NADPH molecules synthesised by photochemistry are used to reduce CO_2_ into sugar precursors through the CBB cycle, where ribulose-1,5-bisphosphate (RuBP) carboxylation is catalysed by RuBisCO and the resulting 3-phosphoglycerate is reduced to glyceraldehyde-3-phosphate (G3P) that is mostly used to regenerate the RuBP used in the CBB cycle. A portion of G3P also serves as a precursor for the synthesis of carbohydrates with myriad functions, including simple sugars (e.g., glucose and fructose), stored energy (e.g., starch), transported energy (e.g., sucrose), structural carbohydrates (e.g., cellulose), amino acids, fatty acids and many other compounds (Paul and Foyer, 2001; Kölling et al., 2015; Wingler, 2018). For each molecule of G3P, three molecules of CO_2_ are assimilated, while nine ATP and six NADPH are consumed during each round of the cycle (Benson et al., 1950; Raines, 2003). CO_2_ assimilation in the chloroplast is dependent on the entry and diffusion of CO_2_ from the atmosphere. Leaf pores known as stomates regulate CO_2_ uptake through changes in stomatal resistance and aperture, and are therefore a major limiting factor for CO_2_ assimilation and plant growth (Lawson and Blatt, 2014; Wang et al., 2014). Stomatal activity responds to changes in light and relative humidity, and is regulated by several coordinated and dynamic signalling mechanisms (Daloso et al., 2017; Devireddy et al., 2018). After entering through stomata, CO_2_ molecules concentrate in the intercellular air space and then pass across the cell wall, plasmalemma, cytosol, and chloroplast envelope before reaching the chloroplast stroma, where they are available to the CBB cycle (Evans and von Caemmerer, 1996; Evans et al., 2009; Tan et al., 2021).

## Photosystem I Photoinhibition: Mechanisms, Impact and Recovery

Although light energy is vital for photosynthetic electron transport, the same energy can damage the photosynthetic machinery when excitation/electron pressure in the photosystem exceeds the capacity of electron consumption by chloroplast sinks. As a result, excitation or electrons are transferred to O_2_, generating reactive oxygen species (ROS) that can oxidise proteins, lipids and metabolites, and can also generate signalling compounds (discussed below). These photo-oxidative conditions are usually triggered by changes in environmental conditions and can lead to a phenomenon known as “photoinhibition,” which is characterised as the inactivation of either or both photosystem(s) (Powles, 1984; Aro et al., 1993; Gururani et al., 2015). Photoinhibition negatively affects photosynthetic capacity and is thus deleterious for plant growth and crop yield (Takahashi and Murata, 2008; Kato et al., 2012; Simkin et al., 2017). Unlike PSII, which is frequently damaged in the light (Tyystjärvi and Aro, 1996), PSI is protected from photoinhibition by several mechanisms (see below). Nonetheless, PSI photoinhibition is induced when PSI is over-reduced, relative to the oxidised state of conventional stromal acceptors, whereupon O_2_ is utilised as an alternative electron acceptor. O_2_ reduction is thought to occur at the PSI acceptor side and/or at the phylloquinone A_1_ site, in each case producing the radical superoxide (O_2_•‒) that is disproportionated to hydrogen peroxide (H_2_O_2_) and O_2_ (Mehler, 1951; Asada et al., 1974; Takagi et al., 2016; Kozuleva et al., 2021). PSI photoinhibition is thought to be the result of the reaction between H_2_O_2_ and the FeS clusters, causing formation of hydroxyl radical (^•^OH) and inactivation of PSI electron transport (Sonoike et al., 1997; reviewed in Sonoike, 2011). Damage to protein subunits by O_2_•‒ and singlet oxygen (^1^O_2_) produced by excitation of O_2_ by triplet P700 (^3^P700) has also been associated with PSI inhibition (Takagi et al., 2016). Notably, the mechanism(s) of ROS production and PSI photoinactivation is not yet fully established.

PSI photoinhibition can be triggered by the combination of light and environmental stresses, such as low temperature, drought and salinity, all of which limit CO_2_ assimilation (Inoue et al., 1986; Terashima et al., 1994; Tjus et al., 1998; Munekage et al., 2008; Takahashi and Murata, 2008). PSI is also susceptible to photoinhibition when the PSI acceptor side capacity is overwhelmed by unregulated electron flow (Munekage et al., 2002; Suorsa et al., 2012; Tiwari et al., 2016; Kanazawa et al., 2017; Lima-Melo et al., 2019a,b) or by various regimes of artificial fluctuating light (Sejima et al., 2014; Kono and Terashima, 2016; Tikkanen and Grebe, 2018). A recent study showed that PSI photoinhibition is intensified in red and blue light, which preferentially excite PSII, when compared with white and green light (Oguchi et al., 2021). In other words, PSI is at risk of inhibition when chloroplast sink capacity is overwhelmed by photosynthetic electron transport activity.

**FIGURE 3:**
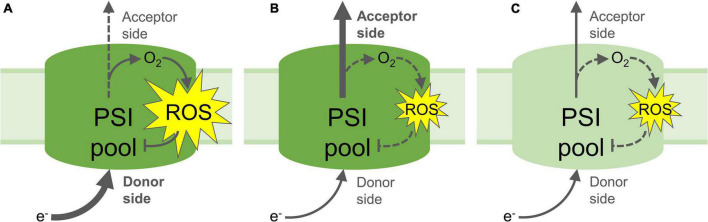
Proposed mechanisms of PSI photoinhibition and photoprotection. **(A)** Increased electron flow to the PSI donor side and/or decreased flow from the acceptor side lead to reduction of O_2_ and formation of reactive oxygen species (ROS), which result in inactivation of part of the PSI pool; **(B)** Non-photochemical quenching, photosynthetic control and/or PSII inactivation down-regulate the flow of electrons to the PSI donor side, limiting the formation of ROS and protecting PSI from photoinhibition. Upregulated electron acceptor capacity in the stroma through increased CO_2_ fixation or photorespiration can also increase flow of electrons from PSI, which may also prevent ROS formation and protect PSI; **(C)** Partial inactivation of the PSI pool by PSI photoinhibition down-regulates transport of electrons to the acceptor side, limiting further PSI photoinhibition and down-regulating electron-consuming reactions in the stroma, including ROS formation and signalling. Yellow stars represent ROS production; Arrows represent electron flow, heavy arrows represent high rate of electron flow, dashed arrows represent low rates of electron flow. Pale green PSI pool **(C)** denotes partial inactivation of the PSI pool through ROS-induced FeS cluster and/or protein damage.

Some studies have demonstrated the negative effects of PSI photoinhibition on CO_2_ fixation, and sugar and starch accumulation, which is attributed to decreased electron transport by a partly inactive PSI pool, and a subsequent decrease in reduced NADPH to power the CBB cycle (Zivcak et al., 2015; Gollan et al., 2017; Lima-Melo et al., 2019a,b). Time-resolved measurements of CO_2_ assimilation and photosynthetic electron transport during the onset and proceeding stages of PSI photoinhibition showed an initial rapid decrease in PSI oxidation and CBB activity, followed by slower rates of decline (Lima-Melo et al., 2019a). These results indicate that the level of PSI inactivation is proportional to the magnitude of energy imbalance between the donor and acceptor sides. Such imbalance decreases in the course of photoinhibition of PSI electron transport, which in turn results in a corresponding decline in the rate of PSI photoinhibition (Figure 3). The negative impact of PSI photoinhibition on CBB activity is particularly acute under low or “growth” light intensities, which are insufficient to fully energise the remaining active PSI centres in order to power stromal reactions (Gollan et al., 2017; Lima-Melo et al., 2019a,b). PSI photoinhibition is especially deleterious to plant fitness due to the fact that the restoration of PSI activity can take a period of days or longer, which is much slower than the mere minutes or hours taken to repair damaged PSII (Kudoh and Sonoike, 2002; Li et al., 2004; Huang et al., 2010; Lima-Melo et al., 2019b). This discrepancy can be explained by the dedicated and efficient PSII repair cycle (reviewed in Aro et al., 1993), while no such repair system for PSI has been identified. Replacement of damaged PSI reaction centre proteins or FeS clusters is widely thought to involve degradation and rebuilding of the entire PSI complex (Scheller and Haldrup, 2005). Nevertheless, PSI recovery appears to be more complex, revealed by employing different methods for evaluating the PSI activity. Decreased abundance of PSI subunit proteins, especially the core proteins PsaA and PsaB and their proteolytic fragments, has been used to demonstrate long-lasting PSI inhibition over several days (Kudoh and Sonoike, 2002; Zhang and Scheller, 2004; Lima-Melo et al., 2019b), while oxidation of P700 and FeS clusters by electron transport appears to recover slightly more quickly (Li et al., 2004; Zhang et al., 2011; Gollan et al., 2017; Lima-Melo et al., 2019b). Rates of CO_2_ assimilation also reflect PSI activity, which is required to provide both reduced NADPH and the proton motive force (PMF) that drives ATP production. Gas exchange measurements showed a more rapid recovery of CO_2_ assimilation after PSI photoinhibition compared to the recovery of PSI activity estimated with analysis of P700 absorbance (Lima-Melo et al., 2019b). Also, the measurement of CO_2_ assimilation revealed that higher light intensity further enhanced the activity of the partly-inactive PSI pool caused by PSI photoinhibition (Gollan et al., 2017; Lima-Melo et al., 2019a,b). These results indicate that electron consumption in the chloroplast may be partly independent from PSI activity and the P700 redox state, and that PSI activity can be enhanced by LHCII-derived excitation and/or activation of “reserve” PSI complexes (Lima-Melo et al., 2019b). Meanwhile, thermal dissipation of excitation energy from LHCII via oxidised P700^+^ in photoinhibited PSI centres (Tiwari et al., 2016; Shimakawa and Miyake, 2019) has prompted the suggestion that photoinhibited PSI does not require replacement at all (Li L. et al., 2018).

## Minimising Photosystem I Photoinhibition Through Photoprotection and Sink Strength

As stated in the previous section, the level of PSI inactivation is proportional to the magnitude of energy imbalance between the donor and acceptor sides, and accordingly distinct mechanisms for PSI photoinhibition avoidance are present at both sides (reviewed in Shimakawa and Miyake, 2018). Mechanisms for protection against PSI photoinhibition at the PSI donor side include inactivation of the PSII reaction centre, dissipation of absorbed light energy as heat and restriction of electron flow through cyt *b6f* (reviewed in Tikkanen and Aro, 2014). Each of these photoprotective strategies down-regulates the flow of electrons to the PSI donor side, reducing electron pressure on the PSI acceptor side and minimising O_2_ reduction. Over-supply of energy to PSII results in the generation of ^1^O_2_ in the PSII reaction centre, which damages the core D1 protein and suspends PSII activity while D1 is replaced (reviewed in Aro et al., 1993; Nixon et al., 2010). This light-induced inactivation of PSII not only relieves the excitation pressure on the remaining PSII complex, but also protects PSI from over-reduction (Tikkanen et al., 2014; Huang et al., 2016). Dissipation of excess excitation from LHCII, known as non-photochemical quenching (NPQ), involves protonation of the PsbS protein and activation of the xanthophyll cycle, both of which are triggered by acidification of the thylakoid lumen (reviewed in Jahns and Holzwarth, 2012; Ruban, 2016). Although NPQ is most often related to PSII photoprotection, NPQ also protects PSI, both directly through quenching part of the LHCII antenna pool functionally associated with PSI (Tikkanen and Grebe, 2018; Hepworth et al., 2021) and indirectly, through down-regulation of PSII activity and relief of electron pressure on the downstream photosynthetic electron transport chain (Han et al., 2010; Sonoike, 2011; Chaux et al., 2015). Aside from activating NPQ, acidification of the thylakoid lumen and subsequent formation of a pH gradient (ΔpH) across the thylakoid membrane also slows proton-coupled electron transport through the cyt *b6f* complex (reviewed in Tikhonov, 2014). This regulation mechanism, known as “photosynthetic control,” is arguably the most important form of protection against over-reduction of both donor and acceptor sides of PSI during sudden increases in light. Plants lacking functional proton gradient regulation 5 (PGR5) protein, which is essential for lumen protonation and thus induction of both NPQ and photosynthetic control, undergo severe PSI inhibition during increases in light intensity (Munekage et al., 2002; Suorsa et al., 2012; Kono et al., 2014; Tiwari et al., 2016; Gollan et al., 2017; Takagi and Miyake, 2018; Lima-Melo et al., 2019a,b). In the *pgr5* mutant, high light-induced PSI photoinhibition is not caused by missing NPQ (Tikkanen et al., 2015; Gollan et al., 2017), demonstrating the importance of photosynthetic control specifically in rapid induction of PSI protection under changing environmental conditions (Rantala et al., 2020).

As PSI photoprotection relies on sufficient acceptor side capacity, improved rates of electron channelling toward strong stromal sinks can alleviate or guard against PSI photoinhibition (Padmasree et al., 2002; Alric and Johnson, 2017; Wada et al., 2018; Yamamoto and Shikanai, 2019). This has been clearly demonstrated through the study of flavodiiron (FLV) proteins, which oxidise electron carriers down-stream of PSI in cyanobacteria, algae, lower-order land plants and gymnosperms, but have been lost from angiosperms (Zhang et al., 2009; Gerotto et al., 2016; Ilík et al., 2017). The introduction of FLV proteins into angiosperm chloroplasts has clearly highlighted the value of stromal sink strength in protecting against PSI over-reduction, which appears to lie at least partly in enhanced electron transport and subsequent lumen protonation, triggering induction of NPQ and photosynthetic control (Wada et al., 2018; Yamamoto and Shikanai, 2019). A major natural electron sink in the chloroplast is the reduction of CO_2_ into sugars through CBB cycle activity. Indeed, a protective effect of elevated CO_2_ concentration against PSI photoinhibition during fluctuating light was recently observed, but was reported to be independent from mechanisms induced by thylakoid ΔpH (Tan et al., 2021). On the other hand, PSI oxidation by photorespiration, which is another major chloroplast electron sink that involves oxygenation rather than carboxylation of RuBP, decreases electron pressure at the PSI donor side by oxidising the electron transport chain (Huang et al., 2015; Osei-Bonsu et al., 2021) and by inducing lumen acidification and photoprotection (Furutani et al., 2020; Wada et al., 2020). Interestingly, weakening of chloroplast sinks in higher plants through down-regulation of CO_2_ fixation has been shown to induce photoprotection mechanisms that minimise PSI photoinhibition (Kohzuma et al., 2009; Joliot and Alric, 2013; Li Y.T. et al., 2018; Wada et al., 2019). Together, these results suggest existence of a regulatory link between CO_2_ assimilation and photosynthetic electron transport that protects PSI from over-reduction, although further research is required to determine how this feedback affects the donor and acceptor sides of PSI.

The prospective role of O_2_ as an electron sink for photoprotection through ROS production has been long speculated, and remains controversial (Ort and Baker, 2002; Miyake, 2010; Driever and Baker, 2011; Ivanov et al., 2012; Cai et al., 2017; Huang et al., 2019, 2021). Although considered not to be a major route for electron flow in leaves (Driever and Baker, 2011), O_2_ reduction by PSI through the so-called water-water cycle (WWC) has been shown to be a genuine mechanism of PSI oxidation (Asada, 2000; Ort and Baker, 2002; Miyake, 2010; Huang et al., 2019). The WWC relies on enzymatic dismutation of O_2_•‒ formed by PSI (Mehler, 1951), followed by detoxification of the resulting H_2_O_2_ in the chloroplast by ascorbate peroxidase (APX) using ascorbate as an electron donor. Ascorbate is regenerated through oxidation of glutathione (GSH), ultimately drawing on the reducing power of NADPH (Foyer and Halliwell, 1976; Foyer and Shigeoka, 2011). Protection from photoinhibition by the WWC has been described to occur through ROS scavenging and electron sink activities (Asada, 1999, 2000; Miyake, 2010), although it appears that the WWC plays only a minor role in PSI protection, depending on the plant species, sample type and the stress conditions studied (Driever and Baker, 2011; Huang et al., 2021). However, the proposed interaction between PSI and the chloroplast antioxidant network, wherein reducing power from PSI both produces ROS and drives ROS scavenging (in which the WWC has an important role), implicates PSI in chloroplast signalling, as discussed below.

## Photosystem I Photoinhibition Impacts Reactive Oxygen Species Metabolism and Chloroplast Signalling

As described above, PSI is a major site of O_2_ reduction and ROS formation in the chloroplast, which not only induces PSI photoinhibition, but also promotes ROS-dependent chloroplast retrograde signalling (reviewed in Gollan et al., 2015; Mullineaux et al., 2018). In particular, the relatively long-lived ROS H_2_O_2_ can move to the nucleus and instigate gene expression through modifying redox-sensitive transcription factors (Exposito-Rodriguez et al., 2017), but can also regulate transcription indirectly by reacting with protein thiol groups or changing the redox state of the antioxidant network (Chan et al., 2016; König et al., 2018; Noctor et al., 2018). Transcriptional reprogramming by H_2_O_2_ signalling is a vital component of both abiotic and biotic stress responses (Vanderauwera et al., 2005; Maruta et al., 2012; Sewelam et al., 2014; Dietz et al., 2016; Smirnoff and Arnaud, 2019; Gollan and Aro, 2020). Relatively low expression of many abiotic stress-responsive genes, which are classical markers for H_2_O_2_ signalling, was observed after PSI photoinhibition (Gollan et al., 2017), indicating a negative impact of PSI photoinhibition on H_2_O_2_ signalling. This was taken to indicate lower levels of O_2_•‒ and H_2_O_2_ in the chloroplast, suggesting that O_2_ reduction is decreased by down-regulated PSI electron transport in a similar way to the decline in CO_2_ assimilation, as discussed above. However, the abundance of O_2_•‒ and H_2_O_2_, along with the activity and expression of ROS-scavenging enzymes, was equivalent in control and PSI photoinhibited leaves after 1 hour of high light stress (Lima-Melo et al., 2019a). This finding indicates that chloroplast H_2_O_2_ deficiency takes place during earlier stages after PSI photoinhibition, or that deficient H_2_O_2_ signalling may have occurred indirectly, such as by decreased photorespiration and subsequently lower H_2_O_2_ production in the peroxisome (Vandenabeele et al., 2004; Sewelam et al., 2014).

In addition to H_2_O_2_ signalling, PSI photoinhibition was also found to suppress production of the oxylipin hormone 12-oxo-phytodienoic acid (OPDA) and down-regulate expression of oxylipin-responsive genes, as well as decreasing the level of lipid peroxidation, during high light stress (Gollan et al., 2017; Lima-Melo et al., 2019a). Oxylipins are products of lipid oxidation, which can occur either enzymatically by lipoxygenase (LOX), or non-enzymatically by ROS, especially ^1^O_2_ and ^•^OH (reviewed in Triantaphylidès et al., 2008; Farmer and Mueller, 2013; Wasternack and Feussner, 2018; Yalcinkaya et al., 2019). Decreased abundance of LOX and OPDA, and lower levels of lipid peroxidation (Gollan et al., 2017; Lima-Melo et al., 2019a), suggest that the enzymatic, rather than ROS-induced, signalling pathway is negatively affected by PSI photoinhibition, although both branches of oxylipin synthesis and signalling pathways appear to be closely interactive (Ramel et al., 2012; Gollan and Aro, 2020). OPDA regulates transcription for biotic and abiotic stress responses, as well as providing a precursor for jasmonic acid (JA), which regulates many stress-responsive and developmental processes (Wasternack and Hause, 2013; Raza et al., 2021). The observed effects of PSI photoinhibition on ROS and oxylipin signalling pathways highlight the importance of PSI activity in transcription regulation, although more work is required in this area to understand the contribution of PSI activity to hormone metabolism and chloroplast signalling.

## Conclusion

PSI electron transport activity directly reduces the stromal electron carrier Fd, leading to both formation of NADPH reducing equivalents, which ultimately support biosynthesis of carbohydrates, and reduction of the thioredoxin network involved in redox regulation of stromal proteins. Although PSI is extremely well protected by regulation of electron flow to the donor side, PSI photoinhibition is induced when insufficient capacity of stromal acceptors leads to ROS formation, causing damage to FeS clusters and/or PSI core proteins; however, ROS formed by PSI over-reduction is an important component of chloroplast signalling and may also have an impact on the redox state of the cellular antioxidant network. The decrease in PSI activity caused by photoinhibition not only down-regulates carbohydrate metabolism, but also negatively affects transcriptional reprogramming through both ROS and metabolic (enzymatic) pathways. This suggests that photoinhibition of PSI during periods of sink weakness may be a mechanism to limit stromal metabolism and ROS formation, preventing excessive reduction of O_2_ and redox-sensitive stromal proteins. Because PSI photoinhibition is mainly avoided by several protective mechanisms, the impact of PSI inactivation on chloroplast metabolism and retrograde signalling seems to be particularly important under specific conditions, such as periods of fluctuating light intensity or low temperature stress. PSI photoinhibition is clearly an expensive option for protection of stromal over-reduction, given the impact on primary metabolism and long recovery time, although prevention of unregulated electron flow to the acceptor side is apparently worth the cost.

## Author Contributions

YL-M and PG devised the work. YL-M, MK, E-MA, and PG wrote the manuscript. All authors contributed to the article and approved the submitted version.

## Conflict of Interest

The authors declare that the research was conducted in the absence of any commercial or financial relationships that could be construed as a potential conflict of interest.

## Publisher’s Note

All claims expressed in this article are solely those of the authors and do not necessarily represent those of their affiliated organizations, or those of the publisher, the editors and the reviewers. Any product that may be evaluated in this article, or claim that may be made by its manufacturer, is not guaranteed or endorsed by the publisher.

## Supplementary Material

Supplementary Video 1 A simplified scheme of the photosynthetic electron transport chain in the thylakoid membrane.
